# Microsurgical posterior fossa re-exploration for recurrent trigeminal neuralgia after previous microvascular decompression: common grounds—scarring, deformation, and the “piston effect”

**DOI:** 10.1007/s00701-023-05877-z

**Published:** 2023-11-13

**Authors:** Gökce Hatipoglu Majernik, Filipe Wolff Fernandes, Shadi Al-Afif, Hans E. Heissler, Joachim K. Krauss

**Affiliations:** Department of Neurosurgery, Hannover Medical School, Carl-Neuberg-Straße 1, 30625 Hannover, Lower Saxony Germany

**Keywords:** Microvascular decompression, Recurrence, Trigeminal neuralgia, Teflon, Jannetta

## Abstract

**Objective:**

Microvascular decompression (MVD) is a well-accepted treatment modality for trigeminal neuralgia (TN) with high initial success rates. The causes for recurrence of TN after previously successful MVD have not been fully clarified, and its treatment is still a matter of debate. Here, we present the surgical findings and the clinical outcome of patients with recurrent TN after MVD who underwent posterior fossa re-exploration.

**Methods:**

Microsurgical posterior fossa re-exploration was performed in 26 patients with recurrent TN (mean age 59.1 years) who underwent MVD over a period of 10 years. The trigeminal nerve was exposed, and possible factors for recurrent TN were identified. Arachnoid scars and Teflon granulomas were dissected meticulously without manipulating the trigeminal nerve. Outcome of posterior fossa re-exploration was graded according to the Barrow Neurological Institute (BNI) pain intensity score. Follow-up was analyzed postoperatively at 3, 12, and 24 months and at the latest available time point for long-term outcome.

**Results:**

The mean duration of recurrent TN after the first MVD was 20 months. Pain relief was achieved in all patients with recurrent TN on the first postoperative day. Intraoperative findings were as follows: arachnoid scar tissue in 22/26 (84.6%) patients, arterial compression in 1/26 (3.8%), venous contact in 8/26 (30.8%), Teflon granuloma in 14/26 (53.8%), compression by an electrode in Meckel’s cave used for treatment of neuropathic pain in 1/26 (3.8%), evidence of pulsations transmitted to the trigeminal nerve through the Teflon inserted previously/scar tissue (“piston effect”) in 15/26 (57.7%), and combination of findings in 18/26 (69.2%). At long-term follow-up (mean 79.5 months; range, 29–184 months), 21/26 (80.8%) patients had favorable outcome (BNI I-IIIa). New hypaesthesia secondary to microsurgical posterior fossa re-exploration occurred in 5/26 (19.2%) patients.

**Conclusions:**

Posterior fossa re-exploration avoiding manipulation to the trigeminal nerve, such as pinching or combing, may be a useful treatment option for recurrent TN after previously successful MVD providing pain relief in the majority of patients with a low rate of new hypaesthesia.

## Introduction

Trigeminal neuralgia (TN) is a clinical diagnosis defined as recurrent unilateral brief electric shock-like pain, with abrupt onset and termination, limited to the distribution of the trigeminal nerve, and triggered by innocuous stimuli [16]. The classical notion of a “simple” neurovascular contact causing TN, introduced in 1932 by Dandy and popularized by Jannetta [19], has been challenged over the years, and possible additional factors have been discussed in its pathophysiology [4, 26, 35].

Microvascular decompression (MVD) is a first-choice surgical option for TN [12], being the only non-ablative procedure that can provide a cure [4], with confirmed high success rates and minimal operative morbidity, especially in TN [3, 27, 36, 43, 44, 47]. Pain recurrence may occur in 47% of patients at 8 years postoperatively [40]. In the largest series with the longest follow-up of MVD for TN, the estimated annual risk of recurrence was about 2 percent over 5 years and less than 1 percent over 10 years [3].

Few studies have been aimed at identifying the risk factors and pathophysiological mechanisms leading to recurrence [1, 2, 9–11, 21, 41, 46, 48]. Possible causes for recurrence might include new vascular contact, scarring/adhesions, and Teflon granuloma, but in some patients, no definitive cause has been ascribed [5, 9, 17, 18, 39, 43]. It is unclear which therapeutic options may be offered to a patient with recurrent TN—either after previous MVD or after ablative surgery. While several studies have suggested percutaneous trigeminal ganglion ablation (by thermocoagulation, balloon compression or glycerol injection), radiosurgery, or peripheral nerve field stimulation [7, 23, 33, 34], few other studies have advocated repeat microsurgical posterior fossa re-exploration.

With regard to the limited data which has become available for surgical re-treatment after previous MVD for TN, we scrutinized the surgical findings and the clinical outcome in a consecutive series of 26 patients with recurrent TN who underwent microsurgical posterior fossa re-exploration according to a standard protocol over a period of 10 years.

## Methods

All patients with a diagnosis of TN who underwent microsurgical posterior fossa re-exploration over a 10-year period at the Department of Neurosurgery, Hannover Medical School, were screened for the present study. Inclusion criteria were a clinical diagnosis of classical drug-resistant TN and recurrence of pain after previous MVD. Patients with a diagnosis of multiple sclerosis were excluded, and data was published elsewhere [15]. Database screening according to the defined inclusion and exclusion criteria resulted in identification of 26 patients. Each patient had magnetic resonance imaging (MRI) before the microsurgical re-exploration.

Departmental assessment methods and surgical techniques for MVD have been described in detail elsewhere [5, 13–15, 29]. All surgeries were performed via a lateral retrosigmoidal approach. Patients were positioned in a modified “semi-concorde” prone position under general anesthesia. The head was flexed and rotated by 45° to the contralateral side. The site of the previous craniotomy was exposed. Then the cranioplasty material if present was removed, if necessary additional bone was drilled to visualize the edges of the sigmoid and the transverse sinus. After opening the dura under the microscope, the cerebellum was gently retracted. Local arachnoid membranes and scar tissue were dissected carefully and completely cut with microscissors to visualize the trigeminal nerve in the depth. The nerve was fully exposed from its entry site to the brainstem to its entrance to Meckel’s cave.

**Fig. 1:**
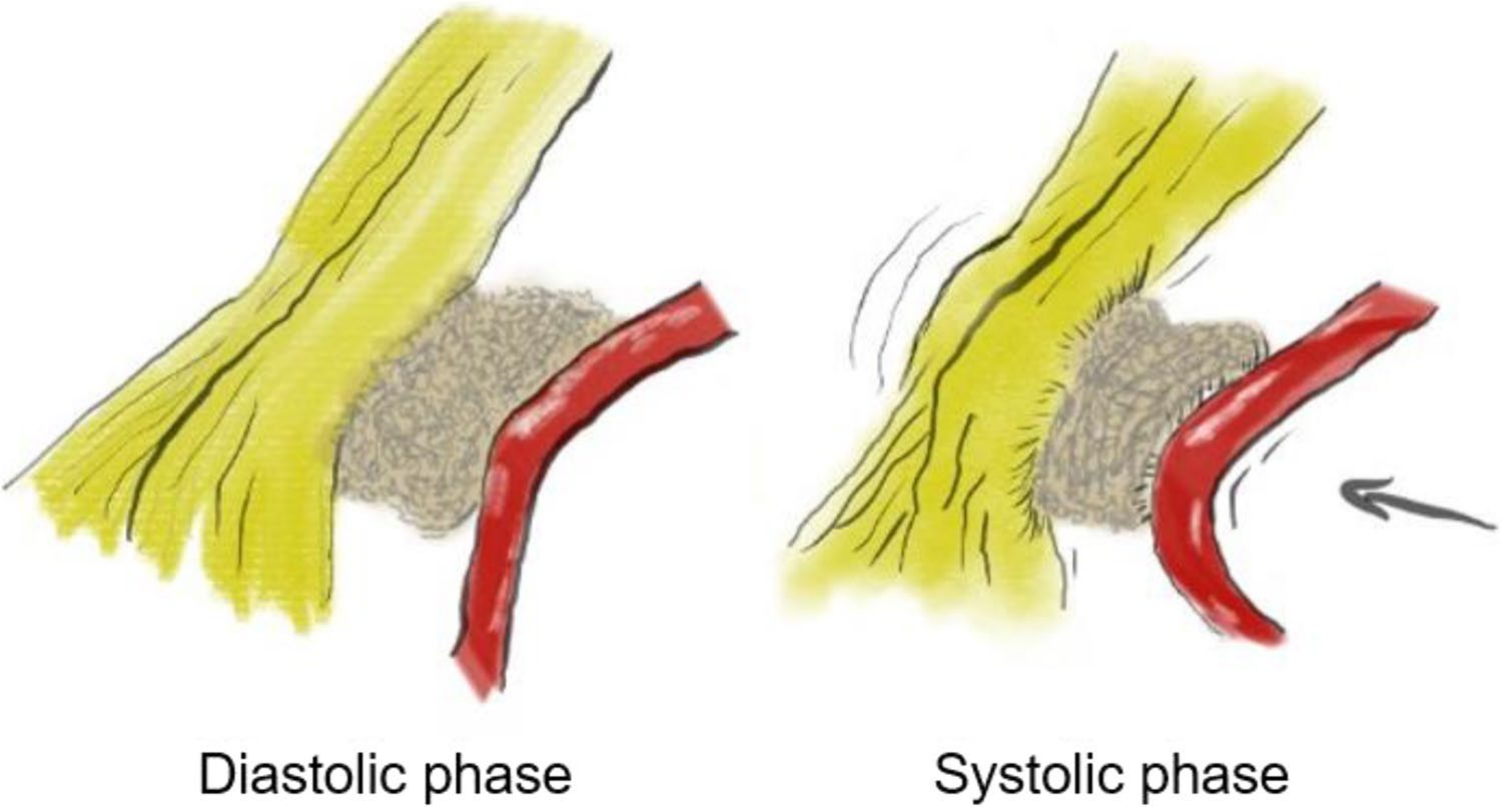
Graphical demonstration of the “piston effect” defined as transmission of pulsations to the trigeminal nerve from systole to diastole from an arterial vessel via the interposed and hardened Teflon

The local topography of the trigeminal nerve was evaluated and assessed for the following findings: dislocation of the Teflon felt, presence of arachnoid scar tissue, evidence of Teflon granuloma, arterial compression, and venous contact. Furthermore, the site was inspected for deformation of the trigeminal nerve and evidence of pulsations transmitted to the trigeminal nerve (without direct arterial contact) through scar tissue or the previously inserted Teflon felt (the “piston effect”). The “piston effect” is defined as transmission of pulsations to the trigeminal nerve from systole to diastole from an arterial vessel via the interposed and hardened Teflon (Fig 1). All arachnoid scars were displayed and dissected meticulously to free the nerve and to resolve its deformity. Veins were dissected and coagulated in the case of venous compression. If Teflon granuloma was detected or the Teflon acted as a transmitter for the “piston effect,” the mass was removed gently. In order to eliminate the “piston effect,” that much Teflon felt was removed until there was no transmission of the pulsations from the artery to the trigeminal nerve. If the artery which had caused trigeminal nerve compression before the first surgery was fixed by scar tissue to the dura, such scars were not dissected. In some patients a new and smaller Teflon felt was inserted as a spacer between the trigeminal nerve and the artery. In no case, the trigeminal nerve was “pinched,” “combed,” or injured in any way. All procedures were performed by the senior author (JKK).

The first clinical evaluation was obtained on the day after surgery. The first regular follow-up visit was scheduled at 3 months after surgery. Patients were followed in the further course either by periodic follow-up visits or by structured phone surveys. Patients were asked about the presence and nature of any facial pain and the severity of residual pain attacks in comparison with the preoperative state, the presence of sensory loss, and any medication for TN. Postoperative outcome was assessed according to the Barrow Neurological Institute (BNI) pain intensity score as adapted from Przybylowski et al.: (I) no pain, no pain medication; (II) occasional pain not requiring medication; (IIIa) no pain, but continued taking medication for fear of stopping; (IIIb) continued pain, adequately controlled with medication; (IV) some pain, not adequately controlled with medication; and (V) severe pain or no pain relief [32]. The 3-, 12-, and 24-month follow-up and long-term follow-up (mean 79.5 months, range 29–184 months) were analyzed. BNI I-III was interpreted as favorable outcome and BNI IV-V as poor.

Statistic evaluation was based on logistic regression analysis. A non-parametric Friedman test was used to compare repeated measures of follow-up scores. Fisher’s exact test was conducted to evaluate differences in outcome with regard to age, gender, side, previous procedures other than MVD, the time between surgeries, symptom duration, and intraoperative identification of the “piston effect.” The JMP®, Version 16 (SAS Institute Inc., Cary, NC), statistical software was used for all analysis. *p*-values <0.05 were considered to be statistically significant.

## Results

### Patients and pain characteristics

There were 13 women (50%) and 13 men (50%). Mean age at the time of surgery for recurrent TN was 59.1 years. The mean intervals between the initial surgery and microsurgical posterior fossa re-exploration are shown in Table 1. All patients had medically refractory classical TN pain attacks (BNI V), and 5 of them (19.2%) reported additionally a permanent pain component. The distribution of pain is presented in Table 2. Five of the 26 patients had mild hypaesthesia detected by the preoperative clinical examination (19.2%), two had complete or partial hearing loss (7.7%), and three presented with ataxia (11.5%).

**Table 1:** Demographic and clinical data of 26 patients with recurrent trigeminal neuralgia undergoing posterior fossa re-exploration after previous microvascular decompression

Patient characteristics	Mean (range)
Age (years)	59.1 (35–82)
Interval between surgeries (months)	59.0 (5–204)
Symptom duration (months)	20.2 (2–94)

**Table 2:** Distribution of pain of 26 patients with recurrent trigeminal neuralgia undergoing posterior fossa re-exploration after previous microvascular decompression

Distribution of pain	No. of patients (%)
Side	
Left	7 (26.9)
Right	19 (73.1)
Topography	
V1	0
V2	4 (15.4)
V3	5 (19.2)
V1 + V2	2 (7.7)
V2 + V3	2 (7.7)
V1 + V2 + V3	13 (50.0)

Four patients also had undergone percutaneous radiofrequency rhizotomy or percutaneous balloon compression of the trigeminal ganglion, and one patient had had gamma knife surgery. In 11 patients, the primary MVD surgery had been performed in another hospital (42.3%), while 15 patients had been operated primarily in the Department of Neurosurgery, Hannover Medical School (57.7%).

### Imaging studies

MRI showed suspected Teflon granuloma in 12 patients (46.1%), suspected neurovascular contact in 3 patients (11.5%), and compression of the trigeminal nerve by a Meckel’s cave electrode for treatment of neuropathic pain in 1 patient (3.8%).

### Intraoperative findings

Recurrent TN was associated with the several findings presented as shown in Table 3. Dislocation of the Teflon felt could not be identified in any instance. In one patient, the trigeminal nerve was compressed by an electrode in Meckel’s cave which had been inserted previously for treatment of neuropathic pain (1/26, 3.8%). Most patients (18/26, 69.2%) had a combination of findings. The patient in whom an arterial compression was identified at the trigeminal entry zone was a 52-year-old man (patient 20). He had had previous surgery in another hospital 70 months earlier. Deformation of the trigeminal nerve could not be assessed unambiguously in all patients during dissection of scar tissue but it was identified to some extent in almost all instances. Also, evidence of pulsations transmitted to the trigeminal nerve through the Teflon inserted previously/scar tissue was clearly identified in at least 15 patients (57.7%).

**Table 3:** Intraoperative findings and measures of 26 patients with recurrent trigeminal neuralgia undergoing posterior fossa re-exploration after previous microvascular decompression

Intraoperative findings and measures (*n*=26)	No. of patients (%)
Intraoperative findings	
Dislocation of Teflon	0 (0)
Arachnoid scar tissue	22 (84.6)
Arterial compression	1 (3.8)
Venous contact	8 (30.8)
Teflon granuloma	14 (53.8)
Cavum Meckeli electrode	1 (3.8)
Piston effect	15 (57.7)
Intraoperative measures	
Total removal of Teflon	9 (34.6)
Partial removal of Teflon	16 (61.5)
Removal of adhesions	22 (84.6)
Removal of electrode	1 (3.8)
Coagulation of vein	5 (19.2)
New Teflon inserted	10 (38.5)

Corresponding to the intraoperative findings several measures were taken (Table 3) tailored to the individual scenario.

### Postoperative outcome and follow-up

There were no severe side effects after microsurgical posterior fossa re-exploration. Postoperative side effects occurred in 8 patients: new sensory deficit in 5 patients (19.2%), from which two cases were transient, transient diplopia secondary to trochlear palsy (3.8%), small asymptomatic cerebellar hemorrhage (3.8%), and delayed facial palsy (3.8%), in one instance, respectively. None of the patients with preoperative partial hearing loss or ataxia had symptom worsening postoperatively.

**Fig. 2:**
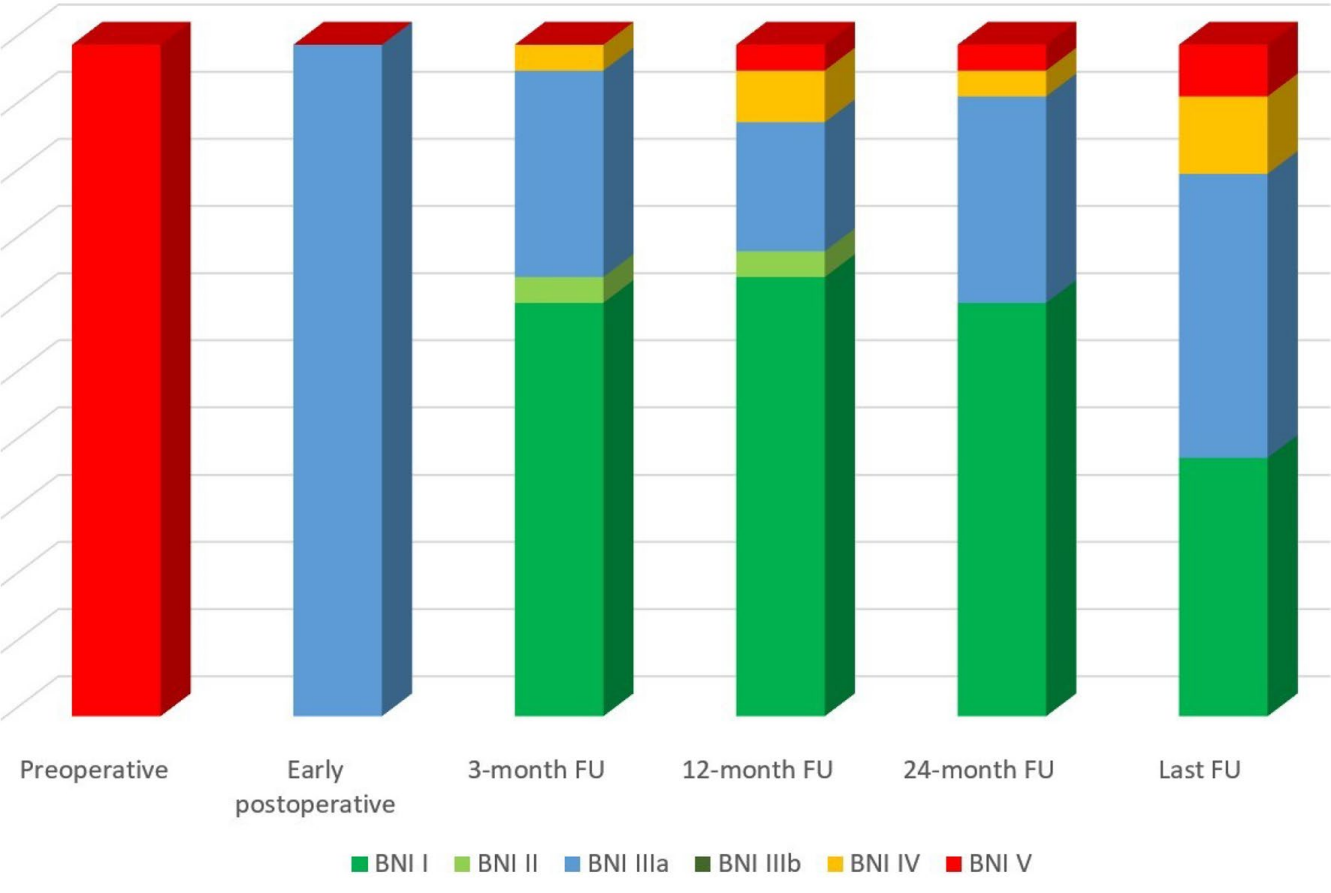
Severity of pain according to the Barrow Neurological Institute (BNI) pain intensity score before and after posterior fossa re-exploration after previous microvascular decompression. Barrow Neurological Institute (BNI) pain intensity score (adapted from Przybylowski et al., 2018): I—no pain, no pain medication; II—occasional pain not requiring medication; IIIa—no pain, but continued taking medication for fear of stopping; IIIb—continued pain, adequately controlled with medication; IV—some pain, not adequately controlled with medication; and V—severe pain or no pain relief. FU: follow-up.

Early postoperative pain relief was achieved in all instances. All patients were available for 24-month follow-up or longer. The individual BNI scores for the different follow-up assessments are shown in Table 4﻿. The distribution of pain scores at the different follow-up periods is shown in Fig. 2. At 3-month follow-up, all patients benefited from pain relief as compared to preoperatively: BNI I 16/26 (62%), BNI II 1/26 (4%), and BNI IIIa 8/26 (31%), and only 1 patient had an unsatisfactory result with BNI IV (4%). At 12-month follow-up, BNI scores were distributed as follows: BNI I 17/26 (65%), BNI II 1/26 (4%), BNI IIIa 5/26 (19%), BNI IV 2/26 (8%), and BNI V 1/26 (4%). The distribution at 24-month follow-up was as follows: BNI I 16/26 (62%), BNI II 0/26, BNI IIIa 8/26 (31%), BNI IV 1/26 (4%), and BNI V 1/26 (4%) (Table 4).

**Table 4:** Longitudinal pain scores of 26 patients with recurrent trigeminal neuralgia undergoing posterior fossa re-exploration after previous microvascular decompression

Patient	Barrow Neurological Institute pain intensity score						
	Before PFRE	Early post operative	3-m FU	12-m FU	24-m FU	Last FU after PFRE	Length of FU after PFRE (months)
1	V	IIIa	IIIa	IV	IIIa	IIIa	60
2	V	IIIa	IIIa	V	V	V	72
3	V	IIIa	I	I	I	IIIa	54
4	V	IIIa	IIIa	IIIa	IIIa	IIIa	44
5	V	IIIa	I	I	I	I	64
6	V	IIIa	I	I	I	IV	135
7	V	IIIa	I	I	I	I	89
8	V	IIIa	I	I	I	I	140
9	V	IIIa	IIIa	I	I	I	93
10	V	IIIa	IV	IV	IV	IV	113
11	V	IIIa	I	I	IIIa	IIIa	44
12	V	IIIa	I	I	IIIa	IIIa	82
13	V	IIIa	I	I	I	I	72
14	V	IIIa	I	I	I	I	87
15	V	IIIa	II	II	I	V	78
16	V	IIIa	I	I	I	IIIa	94
17	V	IIIa	IIIa	IIIa	IIIa	IIIa	169
18	V	IIIa	I	I	I	I	184
19	V	IIIa	I	I	I	IIIa	48
20	V	IIIa	IIIa	IIIa	IIIa	IIIa	92
21	V	IIIa	I	I	I	IIIa	81
22	V	IIIa	I	I	I	I	48
23	V	IIIa	IIIa	IIIa	IIIa	IIIa	60
24	V	IIIa	IIIa	IIIa	IIIa	IV	44
25	V	IIIa	I	I	I	I	29
26	V	IIIa	I	I	I	I	34

Comparison of the individual preoperative, early postoperative (first postoperative day), 3-month, 12-month, 24-month, and the last available follow-up BNI pain intensity scores. Follow-up periods are given as months. Barrow Neurological Institute (BNI) pain intensity score (adapted from Przybylowski et al., 2018): I—no pain, no pain medication; II—occasional pain not requiring medication; IIIa—no pain, but continued taking medication for fear of stopping; IIIb—continued pain, adequately controlled with medication; IV—some pain, not adequately controlled with medication; and V—severe pain or no pain relief

*FU* follow-up, *m* months, *PFRE* posterior fossa re-exploration

At long-term follow-up (mean 79.5 months, range 29–184 months), 10/26 patients (38.5%) had complete pain relief (BNI I), 11/26 patients (42.3%) had pain relief but continued taking medication (BNI IIIa), 3/26 patients (12%) had a BNI score of IV, and 2/26 patients (8%) had a BNI score of V. As compared to 24-month follow-up when only two patients had an unsatisfactory outcome (BNI scores IV and V), there were five such patients on long-term follow-up. The distribution of patients with BNI I-III versus BNI IV-V compared to the preoperative status was statistically significant at all follow-up evaluations (*p*<0.0001). The five patients with poor outcome results on long-term follow-up were offered further surgical treatment, including percutaneous rhizotomy. The exact cause of re-recurrence could not be determined, since none of the patients underwent a new posterior fossa re-exploration. While three patients wanted to wait before making a decision, one patient was lost to follow-up, and another patient was severely disabled secondary to a stroke unrelated to posterior fossa surgery.

### Prognostic factors

The intraoperative presence of arachnoid adhesions significantly predicted favorable outcome (*p*=0.037). Additionally, age was a significant predictor of favorable outcome (*p*=0.037). There was no statistically significant correlation between outcome and gender (*p*=1.00), side (*p*=0.187), affected branches (*p*=0.482), previous procedures other than MVD (*p*=0.920), the time between surgeries (*p*=0.965), symptom duration (*p*=0.757), and intraoperative identification of “the piston effect” (*p*=0.574).

## Discussion

There is no standard treatment for recurrent TN after a previously successful MVD procedure [9]. There are few studies advocating repeat posterior fossa re-exploration in such a scenario; however, these studies are characterized by marked discrepancies regarding intraoperative findings, surgical maneuvers, and length of follow-up [2, 5, 9, 21, 22, 25, 43, 45, 46]. While a recent systematic review has nicely summarized and compared the results of previous studies [20], several questions remain open. Furthermore, there is a paucity of studies comparing posterior fossa re-exploration with other treatment options [8, 43, 48]. Remarkably, according to a prospective study comparing “redo MVD” and percutaneous balloon compression, patients fared better after “redo MVD” considering complications, clinical outcome, and recurrence of TN over a 6-year period [8].

Our study clearly demonstrates that posterior fossa re-exploration in patients with recurrent TN after previous MVD can achieve outcome which is similar to that of patients who underwent MVD for primary TN both with regard to intraoperative complications and to clinical outcome at long-term [36]. Furthermore, we show that the development of recurrent TN may be due to a variety of findings which may act alone or in combination. Taking the individual findings into account will result in patient-specific measures such as removal of scar tissue and Teflon with or without adding new material for decompression. Such personalized approaches also obviate the possible need for additional intraoperative maneuvers to the trigeminal nerve such as combing or partial rhizotomy.

There is quite a high variability in the description of intraoperative findings thought to underly recurrent TN, which may be due to both the surgical technique and the material used during the primary MVD but also to the eye of the beholder during the second surgery. When Jiao et al. tried to summarize the heterogeneous findings in their recent systematic review in 193 of 270 patients, who had repeat surgery for recurrent TN, they reported the following findings: granulomatous lesions or arachnoid adhesions in 31%, vascular conflict in 36%, slippage of Teflon felt in 9%, and no clearly identifiable cause in 29% [20].

Early studies, such as the study published by van Loveren et al. in 1982, predominantly had concentrated on vascular conflicts [42]. In that study, 82% of patients were described to have an aberrant artery or vein in contact with the trigeminal nerve, but only 46% had a significant compression [42]. These findings contrast with newer studies, for example with the study of Chen et al. reporting 50% of cases with Teflon granuloma, 30% cases with new arterial contact, 10% with venous compression, and 10% with negative findings [6]. On the other hand, Cheng et al. reported severe arachnoid adhesions in all their 41 re-operated patients, from which 36.6% also had compression from an artery, 14.6% from a vein, and 19.5% from Teflon [9]. Remarkably, in a recent study from China, incomplete or absent decompression or new vessels were noted in 50% of patients operated for recurrent TN, but all patients also had moderate to severe arachnoid adhesions [43].

Over the past 20 years Teflon granuloma has been identified more frequently as a main cause for recurrent TN [6]. Initially, Teflon had been introduced by Jannetta in 1985 after he had noted that several patients with recurrent TN had stiffened Ivalon implants during repeat surgery which allowed transmission of pulsations to the trigeminal nerve [22]. Teflon was thought to be an inert material with a low rate of tissue reaction [6, 8, 22, 25, 31, 45]. Some studies, however, have shown the migration of multinuclear giant cells in the Teflon material with abundant hyalinized scar tissue with blood supply to the granuloma deriving from the tentorium [5, 6]. While it was suspected that granuloma formation might be triggered by tissue glue that was used to fix the Teflon implant in several studies [5], the findings of our study indicate that it is also the Teflon proper. We considered whether or not to use other materials, such as muscle or Ivalon to pad the vessel-nerve contact, or to use alternatively no-contact sling techniques. Interestingly, a recent multivariate analysis using a 2-center retrospective cohort study of TN patients with MVD using either Teflon or Ivalon found that the implant type had no impact on the final BNI score or the risk of relapse [31].

Arterial pulsations transmitted to the trigeminal nerve through the implanted material which was used for MVD during the primary MVD surgery have received only relatively little attention. We suggest to use the term “piston effect” for this phenomenon since we think that it denotates its mechanisms better than previous rather vague terms or the expression “secondary missile phenomenon” which has been applied by Jannetta to specifically refer to recurrence of TN or hemifacial spasm when using Ivalon [22].

The basis for the “piston effect” most likely is that arachnoid adhesions and granulomatous changes result in hardening of the Teflon, acting as a “glue” uniting the implant with the trigeminal nerve on one side, and the artery on the other. Thus, it appears that two key factors are essential for this phenomenon: hardening of the Teflon and a tight adhesion between nerve, graft and artery. Our study demonstrated that most patients (85%) had arachnoid adhesions or scar tissue at the site of the previous surgery. Indeed, after MVD, the surrounding arachnoid membranes tend to thicken and adhere, altering thus the anatomy of passing structures [35], which may also contribute to compression along the trigeminal entry zone [26], as well as transmission of pulsations from the decompressed vessels [11].

It is quite possible that a high proportion of patients who were described to have unspecific findings upon re-exploration for recurrent TN in early studies had findings as described above or others which were not considered to be possibly causative [18]. Other findings to be considered are distortion or indentation of the trigeminal nerve root [17, 35], which was also noted in our series.

Complication rates for posterior fossa re-exploration after previous MVD vary greatly between different studies. The most commonly reported complication is facial numbness which, however, appears to be related to surgical technique [21, 25, 43, 45, 46]. The frequency of other complications appears to be comparable to that of primary MVD surgery, being less than 5% [20]. According to the systematic review of Jiao et al., the overall occurrence of hypaesthesia is 22% after posterior fossa re-exploration after previous MVD for TN. The frequency of hypaesthesia, however, is markedly higher in series from centers which use mechanical intraoperative measures to alter sensory function of the trigeminal nerve either routinely or in patients in whom no clear cause for recurrent TN has been detected [43]. Such measures include “combing” or “pinching” of the trigeminal nerve or “internal neurolysis,” as well as “partial transection of the trigeminal nerve,” “partial nerve section,” or “partial sensory rhizotomy” [1, 10, 11, 17, 30, 43, 49]. Especially when using more extensive sectioning procedures, facial numbness may occur in more than 50% of patients after posterior fossa re-exploration [1, 31]. On the other hand, the rate of hypaesthesia is remarkably low when using sling methods to keep the offending vessel away from the trigeminal nerve in recurrent TN [20, 46].

Since long-term results for pain relief appear to be similar for “repeat MVD” for recurrent TN regardless of intentional surgical microtrauma to the trigeminal nerve or not, but considering that there is a marked difference in the occurrence of facial numbness, we suggest that such measures may be avoided.

No-contact techniques, such as “sling” or “clip” procedures, or gluing the offending artery to the dura have received more attention recently [24, 25, 28, 37, 38]. Such techniques certainly deserve further consideration both during primary and secondary MVD surgeries.

Limitations of our study include retrospective nature, the lack of standardized preoperative MR protocols, and the lack of a categorical classification system for intraoperative findings, including the degree of trigeminal nerve compression. These limitations, however, are inherent to all studies on this subject. An advantage of our study is that there was no patient attrition and that all patients were available for follow-up of 2 years or longer. We are aware that the results of our study may not be adopted to other scenarios, since all surgeries have been performed by a dedicated senior surgeon. The latter fact, however, made it possible to tailor treatment specifically to intraoperative findings in a more consistent fashion.

## Conclusions

In the present study, we show that microsurgical posterior fossa re-exploration is a useful treatment option for recurrent TN. There were no serious side effects, and the frequency of postoperative hypaesthesia was relatively low. We conclude that microsurgical posterior fossa re-exploration avoiding any intentional damage to the trigeminal nerve is a very useful treatment option in this context. Any maneuvers such as dissecting or “combing” the trigeminal nerve may not be necessary. In the future, it might be worthwhile to explore the more frequent use of sling techniques in posterior fossa re-exploration for recurrent TN.

## Data Availability

The data is available from the corresponding author on reasonable request.
